# Mental Health Review Board Members’ understanding of the policy guideline on 72-hour assessment

**DOI:** 10.4102/curationis.v47i1.2662

**Published:** 2024-11-29

**Authors:** Ontlotlile I. Mpheng, Leepile A. Sehularo, Miriam M. Moagi, Gaotswake P. Kovane

**Affiliations:** 1 Department of Nursing, Faculty of Health Sciences, North-West University, Mafikeng, South Africa; 2 Department of Nursing, Faculty of Health Sciences, University of Limpopo, Sovenga, South Africa

**Keywords:** 72-h involuntary assessment, guideline, mental health, Mental Health Review Board, policy guidelines

## Abstract

**Background:**

The *Mental Health Care Act (No 17 of 2002)* promotes the involvement of Mental Health Review Board (MHRB) members in the oversight, execution and evaluation of assessments and admissions of individuals in accordance with the 72-h policy guidelines. However, the MHRB experiences dissatisfaction with the implementation of policy guidelines on 72-h assessment of involuntary Mental Health Care Users (MHCUs).

**Objectives:**

This study explores and describes the MHRB members’ understanding of the policy guidelines on 72-h assessment of involuntary MHCUs in South Africa.

**Method:**

A qualitative exploratory, descriptive and contextual research design was used. Data were collected using Focus Group Discussions (FGDs) from MHRB from three provinces of South Africa, namely North West, Northern Cape and Gauteng. Three FGDs involving a total of 13 participants were conducted.

**Results:**

Three themes emerged from the data, namely: MHRBs’ understanding of the policy guideline on 72-h assessment of involuntary MHCUs, MHRBs’ challenges with the policy guideline on 72-h assessment of involuntary MHCUs and MHRBs’ recommendations to strengthen the implementation of policy guideline on 72-h assessment of involuntary MHCUs.

**Conclusion:**

Certain issues regarding dissatisfaction related to improper implementation of the 72-h policy guideline persist. Therefore, MHRB recommends that there should be skilled Mental Health Care Practitioners, adequate infrastructure, community involvement, and family and stakeholder collaboration to improve care towards the involuntary MHCUs.

**Contribution:**

The study illustrated there is a need to strengthen the implementation of 72-h assessment of involuntary MHCUs through ensuring enough human resources, designated facilities and involvement of the community as raised by the MHRBs.

## Introduction

The prevalence of mental illness is significantly rising worldwide. Insufficient resources for mental health, inadequate infrastructure and poor service delivery are all challenges to mental health care (Maila, Martin & Chipps 2020). Moreover, substantial evidence has been documented that indicates an increase in mental and substance use disorders as a global concern, impacting individuals, families and communities as one of the leading causes of disability (Collaborators 2019; Vos et al. 2020). These phenomena delay progress on the mental health legislations to ensure efficient service delivery (Docrat et al. 2019). An estimated 6% of people worldwide suffer from severe mental illness, and one in every four households has a family member who has a psychiatric illness (Ndlovu & Mokwena 2023). Ndlovu and Mokwena further share that about 14% of diseases worldwide are caused by mental challenges, leading to at least 1% of mortality. To keep abreast with international human rights standards, the *Mental Health Care Act (MHCA) Act No.* 17 of 2002 introduced the Mental Health Review Board (MHRB) members. The MHRBs are committed as prescribed to check the admission forms filled during the 72-hour assessment of involuntary Mental Health Care Users (MHCUs) in general hospitals before referral to mental health hospitals (Swanepoel & Mahomed 2021). Involuntary MHCUs are individuals that cannot make their own decisions because of their deprived mental health capacity (Swanepoel & Mahomed 2021). To ensure safety and protection of MHCUs during involuntary assessment and admission, MHRB members are expected by the *MHCA (No. 17 of 2002)*, to be the supervisory body who ensure that mental health institutions comply with provisions as stipulated. However in specification, the MHRB members in South Africa (SA) include legal practitioner(s), mental health care practitioner(s) and community member(s).

The 72-h policy guidelines on assessment of involuntary MHCUs in SA were released in 2012 (*MHCA Act No. 17 of 2002*) and they are aimed at ensuring and promoting consistency in assessing involuntary MHCUs for 72-h assessment. The mental health policy guidelines are directed at protecting the rights of MHCUs, and the MHRB members are expected to act as advocates for the MHCUs (Raphalalani et al. 2021). However, the policy guidelines are not appropriately utilised as involuntary MHCUs are at times admitted for more than the expected duration of assessment. To complicate matters further, there is a lack of resources to render proper care to the MHCUs (Raluthaga, Shilubane & Lowane 2023).

When the British Columbia’s *Mental Health Act (MHA)* was changed, the government had the opportunity to adopt changes that would have contributed to closing the admissions accountability gap of MHCUs (Kolar 2018). Notwithstanding the *MHA* in British Columbia, there are still challenges with its application and adherence to regulations for the admission care, and treatment of involuntary mental health cases (Kolar et al. 2022). Additionally, rather than implementing these modifications, the government chose to grant MHRB members, who are also referred to as ‘review panels’, more freedom to devise strategies for assisting long-term MHCUs in reintegrating, overseeing and assessing treatment in accordance with *MHCA*, regulations and policies (Nelson 2021).

In India, the MHRB members include district judge, district collector or district magistrate, psychiatrist and medical professionals. These members are mandated to ensure proper admission, care, treatment and rehabilitation for individuals who are incapable of giving consent or making their own decisions (Gupta, Misra & Gill 2022; Sugiura et al. 2020a). However, it is noted that the legislation in India does not sufficiently address the assessment, care and admission of involuntary mental health units, particularly the lack of resources to offer quality care (Duffy & Kelly 2019; 2020).

In the United States (US), there is an apparent lack of proper assessment, care, treatment and rehabilitation of involuntary MHCUs (Saya et al. 2019). According to Lederer (2022), a mitigation strategy was to involve MHRBs to work hand-in-hand with the mental health community stakeholders, and to act as representatives who oversee professionalism and improve assessment monitoring of the involuntary assessment and admissions. The US civil commitment procedure for involuntary MHCUs is quite improvised as the procedures are not fully complied with; hence, MHRB members are assigned to monitor, review and evaluate compliance in the US (Trivedi et al. 2019). The above-stated information highlights the gap that the care, treatment and rehabilitation of involuntary MHCUs is a global challenge.

In SA, there is improper implementation of the policy guideline on 72-h assessment of involuntary MHCUs during admission, assessment and treatment; there is also a need for close examination by policymakers and experts (professionals) to avoid human rights violations (Freeman & Graham 2022). The challenges leading to improper implementation of the 72-h policy guideline are related to infrastructure where the structures are not conducive to accommodate MHCUs for 72-h assessments, a lack of competent human resources and shortage of medication (Mbedzi 2018). These include limitations at most of the facilities that perform 72-h assessments, as well as the moral and ethical dilemmas with enforcing this evaluation, which is linked to additional patient rights violations. Policy makers should do infrastructural assessment and planning in all facilities designated for providing 72-h assessment, and there should be reviews regarding the policy guideline on 72-h assessment of involuntary MHCUs (Mbedzi 2018). These challenges prompted the researchers to explore and describe MHRB members’ understanding of the policy guideline on 72-h assessment of involuntary MHCUs in SA.

## Research design and method

### Study design

This study followed a qualitative, exploratory, descriptive, contextual research design (Polit & Beck 2021). Such a design allowed the researcher to explore and describe a research phenomenon within a research gap related to the context and literature, and to maintain trustworthiness in this study (Polit & Beck 2021). The breadth and depth of the description derived from a qualitative approach provided a unique insight of the reality of the challenges encountered during the 72-h assessment of involuntary MHCUs.

### Study setting

The data were collected from provincial offices in the three provinces of SA namely, the North West province (NWP), Northern Cape province (NCP) and Gauteng province (GP). Regardless of the challenges encountered in all nine provinces of SA, the three provinces were chosen as they have a historical record of above 90% of inpatient mental health admissions of adults (NCP, 98.7%; NWP, 96.9% and GP, 95.1%) (Docrat et al. 2019). Furthermore, given the high influx of admissions among these three provinces, there is a likelihood of improper implementation of the 72-h policy guidelines. Swanepoel and Mahomed (2021) elucidated that the review boards grapple with practical challenges in ensuring that institutions adhere to the Act’s provisions because of evaluations taking time, shortage of medical staff and lack of resources.

### Study population and sampling strategy

A total of 13 MHRB members between the ages of 43 and 79 years were purposively sampled (Polit & Beck 2021), based on the knowledge of being able to review 72-h assessment and admission documents. Initially, 16 members agreed to participate in the study but 3 were not available during data collection, leaving the study population to comprise 13 MHRB members. The inclusion criteria of this study were males and females appointed in terms of Chapter 4 of the *MHCA Act (No 17 of 2002)*. Participation from the three provinces was as follows: NWP (06), GP (03) and NCP (04). The exclusion criteria were MHRB members, both male and female, who were unavoidably absent or on leave and did not give consent to participate in this study.

### Data collection

After approval was received from the Department of Health (DoH) to conduct the study, an independent MHRB chairperson was assigned per province to recruit participants. The researcher engaged the chairperson through email, telephone and WhatsApp. The MHRB chairperson arranged a virtual meeting between the probable participants and the researcher because of distance between the provinces. During this meeting, the researcher was able to share the study information with the participants and informed them about the independent person responsible for informed consent process. The participants that were interested to participate in the study responded back to the independent person and a date was set for the interview. Semi structured Focus Group Discussions (FGDs) were used to gather rich information from the participants and probing questions were used to clarify responses where necessary (Brink et al. 2016). The FGDs provided an advantage to the participants to stimulate each other’s thinking to provide more information (Brink et al. 2016). Data were collected until saturation. Participants gave permission to record the FGDs. The participant’s cameras were switched on during the interviews, with an assurance of maintaining anonymity when findings of the study are presented. No names were used during data collection and reporting of results. The FGDs lasted between 30 min and 55 min. During the FGDs, the researcher was alone with the participants. A total of 13 MHRB members (Table 1) participated in the three FGDs conducted in this study. The participants were comprised of: six mental health care providers (MHCPs), four Community Representatives (CR) and three Legal Representatives (LR).

**TABLE 1:** Demographic characteristics of empirical phase participants.

Participant	Demographic details of participants		
	Age in years	Occupation in MHRB	Gender
**FGD 1-NWP 06 MHRB members Participated**			
Participant A	60	MHCP	M
Participant B	47	CR	M
Participant C	62	CR	M
Participant D	43	LR	M
Participant E	71	MHCP	F
Participant F	77	LR	M
**FGD 2-NCP 04 MHRB members Participated**			
Participant A	63	MHCP	F
Participant B	71	MHCP	F
Participant C	57	CR	F
Participant D	57	CR	M
**FGD 3-GP 03 MHRB members Participated**			
Participant M	57	LR	F
Participant N	56	MHCP	F
Participant P	79	MHCP	F

Note: Total participants: 13 MHRB members.

FGD, focus group discussion; NWP, North West province; NCP, North Cape province; GP, Gauteng province; MHRB, Mental Health Review Board; MHCP, mental health care provider; CR, community representative; LR, legal representative; M, male; F, female.

Data saturation was reached on the third FGD. The following three main questions were used during the interview schedule:

- What is your understanding of the current practice regarding the policy guideline on 72-h assessment of involuntary MHCUs?
- What is your understanding of the current practice regarding the implementation of policy guideline on 72-h assessment of involuntary MHCUs?
- What can be done to strengthen implementation of policy guidelines on 72-h assessment of involuntary MHCUs?

### Data analysis

Data were transcribed verbatim for analysis. Thematic analysis was conducted following the six-steps defined by Braun and Clarke (2006). The steps include: familiarising self with data, generating codes, finding themes, examining the topics, identifying themes and collating a summary of the findings. An independent co-coder was appointed. The co-coder signed a confidentiality agreement form as a sign of approving to adhere to ethical standards. The researcher and the co-coder analysed the data independently. A virtual meeting was held to discuss the themes and a consensus was reached. After the data were analysed, the researcher shared the information with the MHRB members for the purpose of member-checking.

### Measures of ensuring trustworthiness

A criterion to ensure the trustworthiness of qualitative studies was employed following the credibility, dependability, confirmability and transferability principles (Polit & Beck 2021;1139). Credibility was ensured through extensive prolonged engagement with MHRB. Dependability was promoted by having an audit trail to ensure that the researcher was transparent with steps undertaken from the beginning to the end of the study. Furthermore, a co-coder was utilised during data analysis to verify the findings. Confirmability was ensured by having an audit trail. Moreover, all verbatim transcripts, audio recordings and field notes were given to an independent coder and findings were compared with the researcher’s original analysis and consensus was established after identifying similarities or differences. Transferability was ensured through a thorough description of the research setting, participants and purposive sampling of the MHRB.

### Ethical considerations

The School of Nursing Scientific Committee and the North-West University Health Research Ethics Committee approved this study (Ref: NWU-HREC- 00032-23-A1). Approval was then received from the Provincial Departments of Health of the three provinces (NWP, GP and NCP). Confidentiality and anonymity were maintained by assigning pseudonyms to participants during the interviews. The study information was only accessible to the research team, and all electronic data were password protected and saved on Cloud. All study recordings were kept safe in OneDrive folder, that was only accessible to the research team. Participation was voluntary and informed consent was obtained from those who met inclusion criteria without coercion. Participants were informed of the right to withdraw from the study at any point without being penalised.

## Results

### Themes

Three FGDs were conducted with the MHRB members. Table 2 illustrates the main themes abstracted through the thematic analysis from the FGDs. Each theme contains an outline and sub-themes, followed by quotes.

**TABLE 2:** Themes derived from the three FGDs with the Mental Health Review Board members.

Themes	Sub-themes
1. Mental Health Review Boards’ understanding of the policy guidelines on 72-h assessment of involuntary Mental Health Care Users	1.1 Policy guidelines are used for observation to exclude medical conditions 1.2 Benefits of the policy guidelines on 72-h assessment of involuntary Mental Health Care Users
2. Challenges experienced by Mental Health Review Board in implementing policy guidelines on 72-h assessment of involuntary Mental Health Care Users	2.1 Poor infrastructure 2.2 Administrative challenges 2.3 Challenges related to incorrect or incomplete forms 2.4 Violation of the Mental Health Care Users’ rights 2.5 Shortage of mental health care providers 2.6 Inadequate training of mental health care providers
3. Recommendations for strengthening the implementation of policy guidelines on 72-h assessment of involuntary Mental Health Care Users	3.1 Improvement of the infrastructure 3.2 Recruitment and retention of adequately skilled staff 3.3 Employment of administrative person to check the forms 3.4 Training and development of mental health care providers 3.5 Involvement of the family and community 3.6 Strengthening collaboration among stakeholders 3.7 Amendment of the Act and regulations should be specific about the qualifications of mental health care providers

#### Theme 1: Mental Health Review Boards’ understanding of the policy guidelines on 72-h assessment of involuntary Mental Health Care Users

Two sub-themes emerged from MHRBs understanding of the policy guideline on 72-h assessment of involuntary MHCUs namely, policy guidelines are used for observation to exclude medical conditions and benefits of the policy guidelines on 72-h assessment of involuntary MHCUs. The sub-themes are discussed next.

*Policy guidelines are used for observation to exclude medical conditions*: The MHRB understanding is that involuntary MHCUs under the 72-h assessment are used to exclude medical conditions. The reason is that many MHCUs who are brought in the outpatient department and into the involuntary 72-h assessment come in after having induced substances and other drugs or they might be suffering from an underlying medical condition. To confirm this finding, participants stated:

‘I think the reason for 72-hour observation is to exclude any medical condition, which could have contributed to the confusion of mental status of the patient and the other patient.’ (FGD1, Participant D, M, Legal Rep, 43 years)

‘[*T*]he most important thing is that in terms of the 72-hour policy, if that’s all eeeh general hospitals has to have a 72-hour admission unit because it’s important for us to observe the fact that every mental health care user have a right to be assessed to exclude medical conditions. First before we then send them for 72-hours. So, obviously the 72-hours assessment for us is very important, because it excludes any medical conditions and then the user can then be assessed by a psychiatrist. And from the findings, he will find that that reasonable or he is now presenting with a disorder or mental condition. So in terms of the 72-hours, the user will then be admitted according to the *MHCA* forms that would be eeeh completed by, by the practitioners that would be present in, casualty. So, the act is clear on how we admit.’ (FGD3, Participant A, F, MHCP, 56 years)

*Benefits of the policy guidelines on 72-h assessment of involuntary Mental Health Care Users*: The MHRB stated that there are benefits in the policy guidelines on 72-h assessment of involuntary MHCUs. Mental Health Review Board mentioned that the policy guideline allows the protection of the MHCUs, family members, including members of the community, as the involuntarily admitted MHCUs mostly come in lacking insight of their mental health condition and are a danger to themselves, those around them and the property. The 72-h policy guidelines of involuntary MHCUs stipulate procedures that must be followed during admission and care, human resources needed and the facilities where the involuntary MHCUs are admitted and cared for. This is what the participants said to confirm this finding:

‘There is expectation, and there is what is actually being done. Now the review board is very much updated with the process that must be followed. The guidelines clearly stipulate step by step what is supposed to happen in the 72-hour unit.’ (FGD2, Participant C, F, Community Rep, 57 years)

‘The guideline is very beneficial to our users, except that there is an issue of the implementation, the policy doesn’t have a problem. But the implementation part is our [*most*] serious challenge.’ (FGD3, Participant P, F, MHCP, 79 years)

#### Theme 2: Challenges experienced by Mental Health Review Board in implementing policy guidelines on 72-h assessment of involuntary Mental Health Care Users

Six sub-themes emerged from the MHRBs understanding of the policy guideline on 72-h assessment of involuntary MHCUs namely, poor infrastructure, administrative challenges, challenges related to incorrect or incomplete forms, violation of the MHCUs’ rights, shortage of MHCPs and inadequate training of MHCPs. The sub-themes are discussed next.

*Poor infrastructure*: Mental Health Review Board mentioned that the infrastructures where the 72-h assessment of involuntary MHCUs occurs is not conductive for the type of admission. The safety and accommodation of the involuntary MHCUs are not considered as the MHCUs do not stay in the prescribed infrastructure as stipulated by the *MHCA Act No. 17 of 2002*, and there is often a shortage of beds, culminating in overcrowding. To confirm this finding, participants said:

‘OK, we can. As [*far*] as I am concerned, the psych hospitals are not prepared for admitting patient and looking after the patient when it comes to infrastructure.’ (FGD2, Participant B, F, MHCP, 71 years)

I think there are challenges because I have already alluded that the first thing is that some institutions have no designated unit. That’s the first thing. So, it means infrastructure wise. We are not doing very well (FGD3, Participant N, F, MHCP, 56 years)

*Administrative challenges*: According to the MHRB, there is a lack of administrative personnel in the MHRB to assist in the facilitation of documents to ensure timely review. Availability of human resources needed could also assist in curtailing the breakdown in communication between the MHRB, MHCPs, Head of Health Establishment (HHE) and other stakeholders involved during the processing of documents. Participants affirmed this finding by saying:

‘The reasons or reason could be that The Institution or the establishment would always say we did not have transport to deliver the documents to the review board, or to the department. And the other reason could be that the review board, they could be a Period of time without the secretariat. And the document will be lying here. Without being prepared for the review board.’ (FGD1; Participant E; F, MHCP, 71 years)

‘Can I also add, what we also observed is that there is an administrative challenge because now you expect … now your nurse or your professional staff … what will be empirical because of the absence of a person who manages the document process.’ (FGD2, Participant C, F, Community Rep, 57 years)

In terms of administrative issues? And professional issues, I say administrative because the forms needs to be brought by administrative or secretariat, hence we are having issues (FDG3, Participant M, F, Legal Rep, 57 years)

*Challenges related to the incorrect or incomplete forms*: The MHRB disclosed that incorrect or incomplete filling of forms emerged as a huge problem as they rely on the forms to make a concrete review and decision. If the forms are not properly filled, it means that there is a gap relative to the information needed by the HHE and MHRB, including the court. To confirm this finding, participants stated:

‘With regards mental health practitioners, in completing these forms, that is why I enlarge, as there are a lot of forms that have been returned and some of them are incorrect. Some of them are incomplete.’ (FGD2, Participant C, F, Community Rep, 57 years)

When it gets onto the mental health review board table will sometimes find that the, you know, the birth date of birth is not filled in the. The address is not or. Do you know? Oh, the next of kin, you know, or the applicant (FGD2, Participant A, F, MHCP, 71 years)

‘For me I think and alluded to the forms not being properly completed. As a board, we would make efforts to train although it is not our jurisdiction.’ (FGD3, Participant P, F, MHCP, 79 years)

*Violation of the Mental Health Care Users’ rights*: The MHRB indicated that they were aware of the violation of the MHCUs rights. Violation of MHCUs rights does not only imply physical violation, but just by admitting them in improper infrastructure where their privacy or safety is compromised also constitutes a violation of rights. The MHRB stated that the MHCUs are vulnerable and incapable of making their own decisions; therefore, by not implementing the 72-h policy guideline correctly, this becomes proof of violation of the MHCUs rights. Participants clarified this finding by saying the following:

‘Just to put it simply, there’s non-coherence, non-compliance and a cross violation of the rights of the mental health care users, when it comes to the implementation.’ (FGD2, Participant D, M, Community Rep, 57 years)

‘For my side, the rights that are violated their include … the right to integrity and privacy. As she has already illustrated, the fact that there are institutions that do not have a separate space for them, then you can imagine they’re vulnerable already.’ (FGD3, Participant P, F, MHCP, 79 years)

*Shortage of mental health care providers*: In the mental health setting, MHRB mentioned that there is a lack of MHCPs with specialisation in mental health. The MHRB stated that most challenges, especially the incorrect filling of forms, might be exacerbated by a lack of adequate skills in the MHCPs. The following comments from the participants support this finding:

‘At … at … at most, it’s not about the most difficult ones but most [of] it will be the training of the … of the staff itself, or shortage of the staff itself, but then because of that one, it also [*complicates*] or a situation that is worsened.’ (FGD1, Participant B, M, Community Rep, 47 years)

‘So that even in the specialised in the in the 72-hour institutions we found that there’s no psychiatrist but they have a unit. So, as the board we had to fight with the MEC to hire a psychiatrist there because it’s important that at least the administrative rights of the user is really protected by the specialist there.’ (FGD3, Participant N, F, MHCP, 56 years)

*Inadequate training of mental health care providers*: The MHRB stated that training of MHCPs is taken lightly and most MHCPs require training to ensure adequate care, including correct filling of admission forms. Inadequate training of MHCPs lead to demoralisation because other HCPs claim that they are not trained and do not know what to do in terms of filling forms for admission of the MHCUs. Participants affirmed this finding by saying:

‘As well as, as well as stuffing, people are not well trained, and they don’t have experience to work with this mental health care users.’ (FGD2, Participant B, F, MHCP, 71 years)

‘And so, the implementation in terms of the human resources [*are*] also becoming more problematic because we don’t have the correctly trained people and now we are adding an element.’ (FGD3, Participant N, F, MHCP, 56 years)

#### Theme 3: Recommendations for strengthening the implementation of policy guidelines on 72-h assessment of involuntary Mental Health Care Users

The MHRB made recommendations to strengthen the implementation of policy guidelines on 72-h assessment of involuntary MHCUs. Seven sub-themes emerged namely, improvement of the infrastructure, administrative challenges, recruitment of human resources to check forms, violation of the MHCUs’ rights, recruitment and retention of MHCPs and adequate training and development of MHCPs, involvement of the family and community, and strengthening collaboration among stakeholders.

*Improvement of the infrastructure*: The MHRB members observed a need for improved infrastructure as a priority to meet minimum safety requirements for admission, care, treatment and rehabilitation of the involuntary MHCUs. The infrastructure should meet safety requirements for MHCUs who are violent and aggressive, and to reduce chances of suicides. The space should be sufficient, with enough beds available. During the FGDs, the participants made the following submissions:

‘So, we need to have an … an environment that is really considerate of the condition of the user. So, this infrastructure … very important … and at the same time … sometimes we must be able to observe the user without … maybe being part of them physically … from a distance.’ (FGD1, Participant F, M, Legal Rep, 77 years)

‘… [*B*]ut they’re under the circumstances and the efforts that are made. They are trying to ensure the conduciveness of the environment and to maintain the rights of the users to reduce incidents somewhere.’ (FGD1, Participant B, M, Community Rep, 47 years)

‘I think we must start with the infrastructure and our users should be head to that designated unit and that will cater for their needs. Like the advocate has said they undress they walk naked they also they need that space where you know they we know that they are protected from prying eyes and being judged harshly by the other users who are not mentally ill.’ (FGD3, Participant N, F, MHCP, 56 years)

*Recruitment and retention of adequate skilled staff*: According to the MHRB members who participated in this study, recruitment and retention of adequately skilled staff could result in better handling of the mental health care services in SA as these MHCPs are experienced and have adequate knowledge on what needs to be done. The finding is confirmed by the following statements from the participants:

‘Psychiatric hospitals need staff members who are well trained and experienced, so the hospitals must do the correct thing by appointing the people who understand psychiatry.’ (FGD2, Participant B, F, MHCP, 71 years)

‘So, as the board we had to fight with the MEC to hire a psychiatrist there because it’s important that at least the administrative rights of the user is really protected by the specialist there.’ (FGD3, Participant N, F, MHCP, 56 years)

*Employment of administrative person to check the forms*: Majority of the MHRB members who participated in this study shared that not only a secretariat is needed, but additional administrative personnel is also required to ensure timely facilitation of documents. All documents must be checked and when a form is missing or information is not correctly filled, there is a probability to rectify such gaps during care towards the involuntary MHCUs. Participants emphasised this finding by submitting the following:

‘Training must be done; training must be done … [*it*] must not be done as if it suits somebody. Yeah, training … it’s a problem. There are situations where you find staff strolling in and out of the facilities without training but expected to deliver and comply with the act and go to the board.’ (FGD1, Participant D, M, Legal Rep, 43 years)

‘I must say this because we cannot manage to miss it, the management of these forms, must be corrected like we said earlier on, which includes rechecking counterchecking at that level, but also making sure that all forms are completed, and they are completed properly. But there must be a dedicated person who deals with the forms because these forms must end up at a court.’ (FGD2, Participant C, F, Community Rep, 57 years)

*Training and development of mental health care providers*: Participants recommended that training and development of MHCPs should include mental health workshops, in-service training and being taken to colleges for mental health and specialisation in mental health. Training and development of MHCPs could improve the mental health services provided to the 72-h assessed MHCUs, through improving admission and care forms, proper assessment of MHCPs, informed decision making and being informed advocates for the involuntary MHCUs. Participants confirmed this finding by saying:

‘I think they need to be trained in order mmmh to execute their duties. I think training is needed so that we must be on one page, the review board and the staff. The facilities all must be on one page. I think it is important training.’ (FGD1, Participant A, F, MHCP, 63 years)

‘Now I’m … for me I think … and alluded to the forms not being properly completed. As a board, we must make efforts to train although it is not our jurisdiction.’ (FGD3, Participant P, M, Community Rep, 57 years)

‘They need continuous in-service training to update them and sort of conscientise them.’ (FGD3, Participant M, F, Legal Rep, 57 years)

*Involvement of the family and community*: The MHRB was of the opinion that the MHCUs spend most of their time at home with their families and in the community. They believe that there should be mental health community campaigns, including health education for the family. The family and community members should be provided with in-depth information regarding emergency mental health crises, so that they can actively participate in caring for the MHCUs without exposing them to stigma and discrimination. To confirm this finding, participants said:

‘So [*yeah*], the community must be involved, they must also understand mental illness and that this … ehh … route or referral pathways.’ (FGD2, Participant A, F, MHCP, 63 years)

‘The families who are bringing the users should be given spot information just there that thing can work. Or, if not. We can do what we call early intervention once the user is admitted the family should then be called so that they can be empowered.’ (FGD3, Participant N, F, MHCP, 56 years)

*Strengthening collaboration among stakeholders*: The MHRB mentioned that support is not only required for the MHCPs and the MHRB, but for the other stakeholders too. This includes the South African Police Services (SAPS), psychologists, social workers and the courts. There should be strengthened collaboration to ensure proper understanding regarding the care for involuntary MHCUs. The MHRB observed that other stakeholders involved have no idea regarding engaging in caring for the MHCUs because often the police do not want to assist in restraining the MHCU and taking them to the 72-h assessment facility. This is what participants said to confirm this finding:

‘As professionals we have to work together. For example, we have the SAPS. The South African Police Service. The correctional service. We have … eeeh these are the key people that should work together at all the times. Then we have social services start. And then it goes to the main once in the system the nursing staff, the doctors, and other professionals like psychologists to social workers. Then some senior managers. So, if these people can work together, I think our problems will be resolved.’ (FGD1, Participant B, M, Community Rep, 47 years)

‘SAPS is in the act. The only thing that is problematic is that implementation is very much a problem. The police don’t know where they are actually in these matters. They refuse, will tell you that they are going to burn our cars, you are going to … they are going to break the cars with amatje (stones) you know the stones, so I think the best implementation is that each hospital like … they must reach out to the nearest police station police station and have a relationship with the commander or whoever is in charge, so that when these things happen, they’re able to call the commander and he gives instructions. But if we don’t have a relationship with the police, we’re having problems. I saw it when they’re working very well in the private sector.’ (FGD3, Participant N, F, MHCP, 56 years)

*Amendment of the Act and regulations should be specific about the qualifications of the mental health care providers*: The MHRB recommended that the *MHCA* and regulations including the 72-h policy guideline should be amended to specify the qualifications of the MHCPs. These amendments should provide qualification(s) required by MHCPs to meet expectation according to the guidelines. The HHE, should advocate and/or support the MHCPs in obtaining qualifications required, to ensure that the MHCUs needs are met. Participants affirmed this finding by saying:

‘[*We*] also need a lot of review because it has a lot of gaps and we have identified it as the Gauteng Mental Health Review Board. We have a lot of gaps in the *Act* that need to be tightened up, so the human resource is really not satisfactory because most of the people are not qualified to do the work that they are doing.’ (FGD3, Participant N, F, MHCP, 56 years)

## Discussion

### Mental Health Review Board understanding of the policy guidelines on 72-h assessment of involuntary Mental Health Care Users

The MHRB indicated that policy guidelines are used for observation to exclude medical conditions. This finding is consistent with those of Wilson et al. (2023) who indicate that to ensure that the MHCUs mental breakdown is not because of a medical condition, the 72-h policy guideline prescribes that the MHCUs be observed for a maximum of 72-h to exclude prior medical conditions. However, the benefits of the policy guidelines on 72-h assessment of involuntary MHCUs go beyond excluding medical condition as shared by the MHRB. As stated in the *MHCA (Act No. 17 of 2002),* the benefit of the 72-h policy guideline is to ensure care, treatment and rehabilitation for involuntary MHCUs, considering special procedural guides which guard against discrimination and rights violations.

### Challenges experienced by Mental Health Review Board in implementing the policy guidelines on 72-h assessment of involuntary Mental Health Care Users

The MHRB indicated that the conditions at facilities where involuntary MHCUs are admitted are poor and do not meet the requirements for the admission of involuntary MHCUs. This finding is supported by Essien and Asamoah (2020) who indicate that there is poor mental health infrastructure where the MHCPs are admitted and the structures do not meet the requirements to accommodate the MHCUs (Essien & Asamoah 2020). Another issue is administrative challenges that persist in mental health settings. To confirm this finding, Vanagundi et al. (2023) indicate that there are administrative processes that are unclear procedurally, and in the context of documentation, admission, care, treatment and rehabilitation. The MHRB also recognises the challenges related to the incorrect or incomplete forms that are often returned to them from the high court. This finding is supported by Frost et al. (2023) who state that there are delays in mental health emergency paper records, making mental health emergency care and continuity of care difficult. These authors postulate that, even in cases where records are made accessible, information is frequently lacking and records are incorrect.

According to the MHRB who participated in this study, there is a serious problem of violation of the rights of MHCUs in SA. For instance, incorrect forms were received, and this constitutes rights violation (Buckingham 2018). This is in contradiction to Chapter 2 of the *Constitution of the Republic of SA* which advocates for the respect of rights of all people who should receive health care services in the country. The overcrowding of MHCUs in South African mental health care institutions is an ongoing concern raised by participants, and this conundrum is also recognised by the South African Human Rights Commission (SAHRC 2019). The lack of human resource leads to the misdiagnose of MHCUs, their relapsing, leading to their continuous admission (Johnson, Drescher & Bordieri 2023; Malakoane et al. 2020; Murphy et al. 2022). The MHRB also raised concerns regarding inadequate training of MHCPs who care for involuntary MHCUs. These findings corroborate with those of Essien and Asamoah (2020) who mention that there is a poor development programme and inadequate training for MHCPs.

### Strengthening the implementation of policy guidelines on 72-h assessment of involuntary Mental Health Care Users

Participants made recommendations that could be used to strengthen the implementation of policy guidelines on 72-h assessment of involuntary MHCUs. They proposed that improvement of the infrastructure would enhance mental health service delivery to the involuntary MHCUs. They supported that improvement of infrastructure accessibility to the mental health care should be a priority and this will ultimately be conducive for the MHCUs (Darmawan et al. 2023; MacGregor, Brown & Stavert 2019; Szabo & Kaliski 2017). For satisfactory facilitation of involuntary care and treatment and rehabilitation of the MHCUs, the admission process should include a family member, qualified professional nurse, medical doctor or a psychiatrist, clinical psychologist, social worker, MHRB and the HHE. Additionally, the court must ensure that the rights of the MHCUs are protected and that the MHCUs require admission under involuntary MHCUs (Wu 2023). This information indicates there are many policy gaps and a lot still needs to be done to improve the implementation of policy guidelines on the 72-h assessment of involuntary MHCUs.

For the smooth flow of administration and admission documents, there is a need for employing an administrative person to check the proper completion of documents. According to the *MHCA (Act No. 17 of 2002)*, the MHRB must receive the appeal, through its secretariat, and ensure that all documents are obtained from the HHE within 30 days of receiving the notice of appeal and are delivered to the review board members no later than 1 week before the appeal. However, this becomes a challenge because at times the forms arrive late to the HHE especially if there are no personnel to verify the documents, including the collection of documents in time, which may inconvenience the MHRB in making a concrete decision on time (Malakoane et al. 2020). These delays in the submission of documents to the MHRB, leave the MHRB frustrated, hence the need to have a secretariat for that purpose.

The MHRB who participated in this study support the training and development of MHCPs for purposes of competency. This finding is confirmed by the recent studies of Houton et al. (2022) and Murphy et al. (2022), who indicate that in-depth training should be provided to all MHCPs included in caring for the 72-h assessment of involuntary MHCUs. On training and development related to further care with the MHRB, there should be involvement of family members during care, treatment and rehabilitation of the MHCUs. A matter of concern is that some of the family members are only present during admission of the MHCUs, they disappear during admission or are unable to come to the health facility because of challenges related to distance when MHCUs are admitted far from their homes (Sugiura, Pertega & Holmberg 2020b). Additionally, the MHRB recommends that the reformation and implementation of community mental health services, including education of the community members and those around the MHCUs must ensure protection of the MHCUs rights and enhance the insight of the community (WHO 2021a, 2021b). Furthermore, there is need for implementation of community out-reach programmes to share information regarding mental health and its impact on policy decisions.

The participants shared that there must be strong collaboration among stakeholders. The participants further stated that they realised miscommunication and lack of knowledge from the SAPS. For an example, in instances where the involuntary MHCU is violently aggressive (verbally and physically), Section 40 of Chapter 5 of the *MHCA No. 17 of 2005,* permits the SAPS to be in charge of an MHCU’s welfare in a prehospital environment (Barbui et al. 2021; Gilhooley et al. 2017; Stander, Hodkinson & Dippenaar 2021). However, some of the SAPS personnel refuse to assist the MHCUs because of the lack of knowledge of their responsibilities as stipulated in the *MHCA*. On the other hand, the HHE must ensure that the admitted involuntary MHCU receives appropriate mental health services, MHCPs evaluate the user’s physical and mental health status for 72 hours in the way specified, and making sure that the MHCPs also consider whether the involuntary services need to be continued (*MHCA No. 17 of 2002*).

### Recommendations

The MHCPs and MHRBs must be trained and developed to ensure their expertise in 72-h admission document facilitation. Their training must include continuous workshops and in-service training for correctly filling the *MHCA* forms. The MHRB members must always be involved in workshops related to mental health including the 72-h assessment trainings, to allow them to gain knowledge and adjust to developing strategies and measures used to improve provision of care in mental facilities. Furthermore, the Act and the regulations should establish the credentials of mental health practitioners caring for involuntary MHCUs. The *MHCA* must clearly specify requirements for MHCPs to care for involuntary MHCUs. The head of a health establishment must advocate for appropriate 72-h designated health facilities, in relation to space, privacy, good ventilation, lighting and acquired safety.

## Conclusion

In conclusion, there should be accessible mental health services during the provision of care to the involuntary MHCUs under the 72-h assessment admission, treatment and rehabilitation. Furthermore, to ensure proper facilitation of the *MHCA* and the 72-h policy guidelines, there must be a secretariat for delivery of documents including MHCPs who are adequately trained and have specialisation in psychiatry.
