# Corrigendum: Mental health review board members’ understanding of the policy guideline on 72-hour assessment

**DOI:** 10.4102/curationis.v47i1.2738

**Published:** 2024-12-19

**Authors:** Ontlotlile I. Mpheng, Leepile A. Sehularo, Miriam M. Moagi, Gaotswake P. Kovane

**Affiliations:** 1 NuMIQ Research Focus Area, Faculty of Health Sciences, North-West University, Potchefstroom, South Africa; 2 Lifestyle Diseases Research Focus Area, Faculty of Health Sciences, North-West University, Mahikeng, South Africa; 3 Department of Nursing, Faculty of Health Sciences, University of Limpopo, Sovenga, South Africa

In the published article, Mpheng, O.I., Sehularo, L.A., Moagi, M.M. & Kovane, G.P., 2024, ‘Mental health review board members’ understanding of the policy guideline on 72-hour assessment’, *Curationis* 47(1), a2662. https://doi.org/10.4102/curationis.v47i1.2662, there was an error in affiliation 1.

Instead of:

Department of Nursing, Faculty of Health Sciences, North-West University, Mafikeng, South Africa

For the 1st and 4th authors (Mpheng & Kovane), it should be:

NuMIQ Research Focus Area, Faculty of Health Sciences, North-West University, Potchefstroom, South Africa

For the 2nd author (Sehularo), it should be:

Lifestyle Diseases Research Focus Area, Faculty of Health Sciences, Mahikeng, South Africa

The authors apologise for this error. The correction does not change the study’s findings, its significance or overall interpretation of its results or the scientific conclusions of the article in any way.

